# Vibrational behavior of psyllids (Hemiptera: Psylloidea): Functional morphology and mechanisms

**DOI:** 10.1371/journal.pone.0215196

**Published:** 2019-09-11

**Authors:** Yi-Chang Liao, Zong-Ze Wu, Man-Miao Yang

**Affiliations:** Department of Entomology, National Chung Hsing University, Taichung City, Taiwan; University of Saskatchewan College of Agriculture and Bioresources, CANADA

## Abstract

Vibrational behavior of psyllids was first documented more than six decades ago. Over the years, workers have postulated as to what the exact signal producing mechanisms of psyllids might be but the exact mechanism has remained elusive. The aim of this study is to determine the specific signal producing structures and mechanisms of the psyllids. Here we examine six hypotheses of signal producing mechanisms from both previous and current studies that include: wingbeat, wing-wing friction, wing-thorax friction, wing-leg friction, leg-abdomen friction, and axillary sclerite-thorax friction. Through selective removal of possible signal producing structures and measuring wing beat frequency with high speed videos, six hypotheses were tested. Extensive experiments were implemented on the species *Macrohomotoma gladiata* Kuwayama, while other species belonging to different families, i.e., *Trioza sozanica* (Boselli), *Mesohomotoma camphorae* Kuwayama, *Cacopsylla oluanpiensis* (Yang), and *Cacopsylla tobirae* (Miyatake) were also examined to determine the potential prevalence of each signal producing mechanism within the Psylloidea. Further, scanning electron microscope (SEM) was used to examine possible rubbing structures. The result of high speed video recordings showed that wingbeat frequency did not match the dominant frequency of vibrational signals, resulting in the rejection of wingbeat hypothesis. As for the selective removal experiments, the axillary sclerite-thorax friction hypothesis is accepted and wing-thorax friction hypothesis is supported partially, while others are rejected. The SEM showed that the secondary axillary sclerite of the forewing bears many protuberances that would be suitable for stridulation. In conclusion, the signal producing mechanism of psyllids may involve two sets of morphological structures. The first is stridulation between the axillary sclerite of the forewing and the mesothorax. The second is stridulation between the axillary cord and anal area of the forewing.

## Introduction

Vibrational communication is prevalent among the insects with more than 18 orders having been recorded to communicate via substrate-borne signals [1]. Vibrational signals of insects usually play an important role in mating behavior [2–4]. Other functions of vibrational signals also include defense [5] and food searching [6–8]. In Hemiptera, the mechanisms of vibrational signal producing vary greatly, for example, members of Heteroptera (bugs) can emit vibrational signals by stridulation, tymbal buckling, or abdomen vibration, while many species of Auchenorrhyncha (cicadas, planthoppers, leafhoppers, treehoppers) utilize a tymbal organ to produce vibrational signals [9]. Additionally, whiteflies (Sternorrhyncha, Aleyrodoidea) produce vibrational signals through abdominal oscillation [10] and aphids (Aphidoidea) emit vibrational signals by rubbing the abdomen and hind legs against substrates [11]. As well, psyllids (Psylloidea) are comparatively active singers during their mating behavior but until now their mechanisms of signal production have been poorly understood [12–16].

The vibrational behavior of psyllids was first reported by Ossiannilsson in 1950 [17]. He suggested that psyllids produced faint signals via wing vibration. In 1952, Tuthill [18] suggested that the radula of the forewing may hold the potential for stridulation and Heslop-Harrison [19] proposed that stridulation occurred between the inner side of legs and the bee-hive-like structure of the abdomen. He also suggested that there was potentially a second signal producing mechanism in psyllids, via leg-leg stridulation through examination of members of 85% of the known psyllid genera from all geographic areas, he found that psyllids did not possess a tymbal-like structure [20]. Taylor [21, 22] discovered sclerotized structure on the second anal vein of both wings and scale-like denticles on the axillary cords of the meso- and metascutellum of psyllids. He suggested psyllids generate vibrational signals by stridulation between these two anatomical structures. Tishechkin [23] examined the thoracic characteristics of 14 species among four families of psyllids and agreed with the hypothesis of Taylor [21, 22]. Wenninger et al. [24] stated that the spectral pattern of *Diaphorina citri* Kuwayama (Liviidae) vibrational signals is similar to acoustic signals produced by wingbeats of small flying insects. He and his colleagues postulated that vibrational signals in psyllids were generated partly by wingbeats. If true, it would also be possible that psyllids could solely use rapid wingbeats to produce signals. More recently, Eben et al. [25] agreed that psyllids produce signals by stridulation between the axillary cord of the thorax and anal area of the forewings. Despite all the work and hypotheses and partly due to the small size and difficult observation of these taxa, the exact mechanism has remained speculative and unclear.

Based on previous studies of the potential signal producing mechanisms of psyllids, we have identified six hypotheses to be examined. The description of these six hypotheses are as follows: (1) wingbeat hypothesis [17]: psyllids make signals via wingbeats without any friction between structures; (2) wing-wing friction hypothesis [18, 20]: psyllid forewings possess a tooth-like structure so as to rub each other when psyllids vibrate their wings; (3) wing-leg friction hypothesis: this hypothesis was also proposed by Heslop-Harrison [20]; (4) wing-thorax friction hypothesis [12, 21, 22, 23, 25]: forewing rubs against the thorax when wings vibrate. Specifically, friction occurs between the anal area of the forewing and the axillary cords located on the meso- and metascutellum; (5) leg-abdomen friction hypothesis [19]: abdomen contraction can be seen when psyllids emit signals, therefore, Heslop-Harrison thought that the friction occurs between the inner side of the leg and abdomen, of which, the first sternite possess a rough bee-hive-like structure; (6) axillary sclerite-thorax friction hypothesis: this is a new hypothesis that is first proposed in this study. We suggest here that the axillary sclerite makes contact with the thorax while the forewings vibrate rapidly and that is how psyllids produce vibrational signals.

Most of the current hypotheses involve wings and wing movement for signal production, hence, specific experiments are designed to verify each hypothesis and examine the most likely mechanism. These experiments are implemented through wing-cut and observation, through high-speed video recordings of the wingbeat when psyllids emit signals and by taking scanning electron microscope (SEM) images to demonstrate the existence of specific anatomical structures that might serve in signal production.

## Materials and methods

### Ethics statement

The field work of sample collecting in this study has obtained the permission from Forestry Bureau, Council of Agriculture, Executive Yuan (Permit numbers: Hsinchu-1022101424, Dongshih-1023100925, Dongshih-1033100710, Nantou-1024101185, Taitung-1037150568).

### Insect preparation

Psyllid larvae were collected from the field (S1 Table) and positioned in an incubator maintained in a dark environment at a temperature at 25±2°C. Because one psyllid species, *D*. *citri*, has been studied only mates during the daytime instead of at night, we assumed that similar behavior occurred in our studied species [26]. Therefore, psyllids were held in darkness to discourage mating and we believed that this pretreatment will encourage calling behavior of psyllids when ready for observations. However, mating may have not been completely prevented by this treatment and so we cannot ensure that all tested individuals maintained their virginity. Larvae were raised within a plastic box (27 x 19 x 5 cm) in which plant shoot cuttings were inserted in a vial of water. Emerging adults were isolated individually into a small plastic box (15 x 8 x 5 cm) and sexed immediately after eclosion.

Extensive experiments were implemented on the species *Ma*. *gladiata* (Homotomidae). This species was originally widespread in the Orient and South Asia but has become an invasive pest in Europe and USA [27–31]. The vibrational behavior of this species has been described in detail [15]. Body size of *Ma*. *gladiata* is comparatively large (body length including forewing is about 5–6 mm) and this species actively makes duet signals, which makes it a good model species to be observed and conduct serial experiments on.

**Fig 1:**
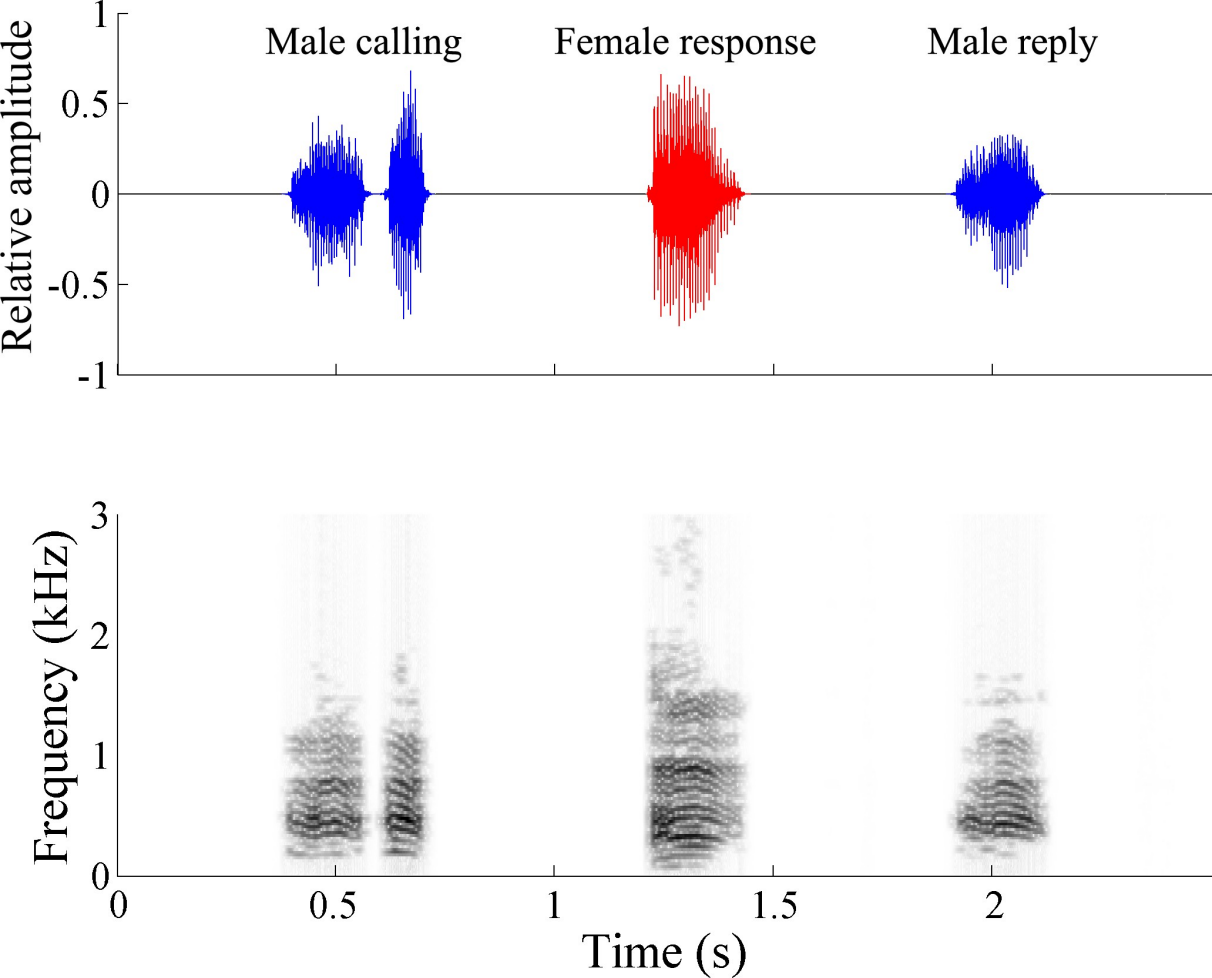
The oscillogram (top) and spectrogram (bottom) of vibrational signals of *Macrohomotoma gladiata* Kuwayama. Blue: male, red: female.

In the mating behavior of *Ma*. *gladiata*, the male produces a two-chirp call and the female replies with a single chirp, after that, the male responds with a single chirp and then begins searching behavior (Fig 1, S1 Audio) [15]. As vibrational signals of psyllids differ among different taxa even in closely related species, different species may possess different signal producing behavior [13–15]. Other species belonging to different families, i.e., *T*. *sozanica* (Triozidae), *Me*. *camphorae* (Carsidaridae), *C*. *oluanpiensis* (Psyllidae), and *C*. *tobirae* (Psyllidae) were also examined to determine the prevalence of signal producing and the associated mechanisms within the Psylloidea. *Me*. *camphorae* is widespread in low elevations of Taiwan and feeds on *Hibiscus* and *Urena* spp. (Malvaceae); *T*. *sozanica* is a pit gall inducer that is specific to *Daphniphyllum* spp. (Daphniphyllaceae); *C*. *oluanpiensis* and *C*. *tobirae* feed on *Pittosporum pentandrum* (Blanco) Merr. and *Pittosporum tobira* Ait., respectively. The latter two species of *Cacopsylla* have been described in detail for their species properties and vibrational behavior [14].

### Recording of vibrational signals

Vibrational recording of psyllids mainly follows the methods of Liao and colleagues [13, 14]. Recording was conducted in an anechoic chamber illuminated with a 2-foot long fluorescent tube. In each trial, a single psyllid individual (usually male) was gently settled on a plant shoot which confined in a plastic tube (15 x 5 cm) to restrict psyllids from jumping away. Vibrational signals were received by a gramophone stylus which slightly touched the base of the plant shoot and was then amplified through an amplifier (Lzban, DRA-455, China) and saved in a dictaphone (Laxon, USB-F20, Taiwan). If psyllids did not make any signals for a while, we played back recorded conspecific signals to induce vibrational behavior by using a dictaphone (Sony NWZ-E435, Japan). The dictaphone was positioned near the base of plant shoot without direct contact and played the recorded signals every 5 seconds. The air-borne signals from the dictaphone induced vibrational signals in the plant [32]. Sampling rate for signal recording was 48,000 Hz and bit depth was 32-bit resolution.

### Wing-cut experiments

**Fig 2:**
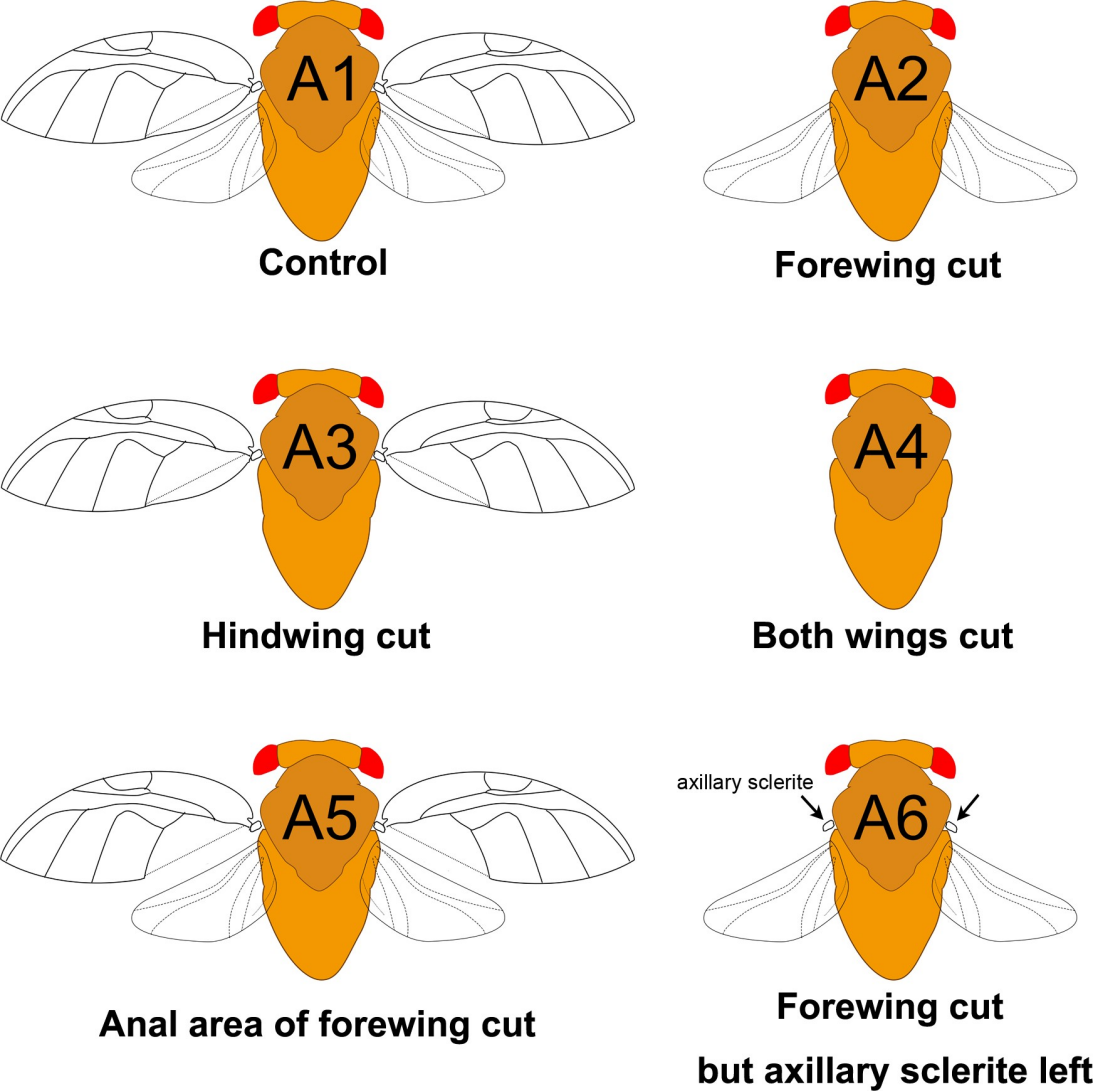
Illustration of each treatment of wing-cut in the study.

We used a series of wing-cut experiments to examine the role of wings in signal production (Fig 2). The treatments are A1: control, without any treatment; A2: forewing cut; A3: hindwing cut; A4: both wings cut; A5: anal area of forewing cut (S1 Fig); A6: forewing cut but axillary sclerite left. Adults were immobilized by chilling on a cold pack. Specific parts of wings were removed by a customized scissor. Psyllids that had been treated were settled in the same rearing area for at least one night before recording was attempted.

*Macrohomotoma gladiata* was used in all six treatments. Other psyllid species belonging to different families were used to confirm the prevalence of certain mechanisms. We selected six individuals from the other four species representing 3 families for further examination, i.e., *T*. *sozanica*, *Me*. *camphorae*, *C*. *oluanpiensis*, and *C*. *tobirae*. First, we recorded their original signals as a control treatment (A1). After that, three individuals of each species were treated by cutting the forewing (A2), while the other three individuals were treated by cutting the forewing but leaving the axillary sclerite intact (A6). Then, we recorded the vibrational signals of the individuals of A2 and A6 of these four species again and compared their signals with A1.

### Wingbeats recording

A high-speed camcorder (GC-PX100 BUS, JCV, Canada) equipped with 8X microlens was positioned and focused on a male to observe its call in the control situation (A1). This experiment was only completed on a single individual of *Ma*. *gladiata*. Through high-speed video recorder (600 frames per second), we were able to count the number of *Ma*. *gladiata* wingbeats when it produced vibrational signals.

### Statistical analysis and plotting

We picked up two to three complete vibrational calls from each individual for analysis. The signal processing and statistical analysis was conducted by using Matlab 8.0 (R2012b, Mathworks, Natick, MA). For audio file reading we used scripts by Ellis [33]. The script for noise reduction and plotting was modified from Vincent [34] and Zhivomirov [35]. We obtained the amplitude of signals by using Matlab in which the value of amplitude is normalized within the range -1 to 1 that correspond to the minimum and maximum voltage that can be produced by the electronic circuit of recorder. The value of signal amplitude shown in this study is a ratio. Because the recording equipment and setup were fixed in this study, therefore, the voltage ratio of signal from different treatments can be directly compared. The signal amplitude of psyllids from different treatments was compared using Wilcoxon signed-rank test and Kruskal-Wallis test by Matlab 8.0 with Statistics Toolbox 8.1. Plot was made via Sigma Plot 10.0 (Systat Software, San Jose, CA).

### Scanning electron microscopy

Psyllid specimens previously used for recordings of vibrational signals were preserved in 70% ethanol. The specimens were dried in an oven at 42 degree for at least 10 hours. Each individual was dissected to examine the dorsal and lateral view of the thorax and the right axillary sclerite to facilitate this, the head, wings, legs, and abdomen were removed. Specimens were mounted on standard card stock and coated with gold via a sputter coater (Polaron SC502, UK). All images were taken with a scanning electron microscope (SEM) (Topcon ABT-150S, Japan) located at the Department of Plant Pathology, National Chung Hsing University, Taichung, Taiwan.

## Results

### The wingbeat frequency

We measured the wingbeat frequency of one male of *Ma*. *gladiata* when it produced signals. The video (S1 Video and S2 Video) showed that each chirp is composed of many continuous fast wingbeats at 118.32 ± 5.68 Hz for the first chirp and at 139.97 ± 4.18 Hz for the second chirp (S2 Table).

### Wing-cut treatments

**Fig 3:**
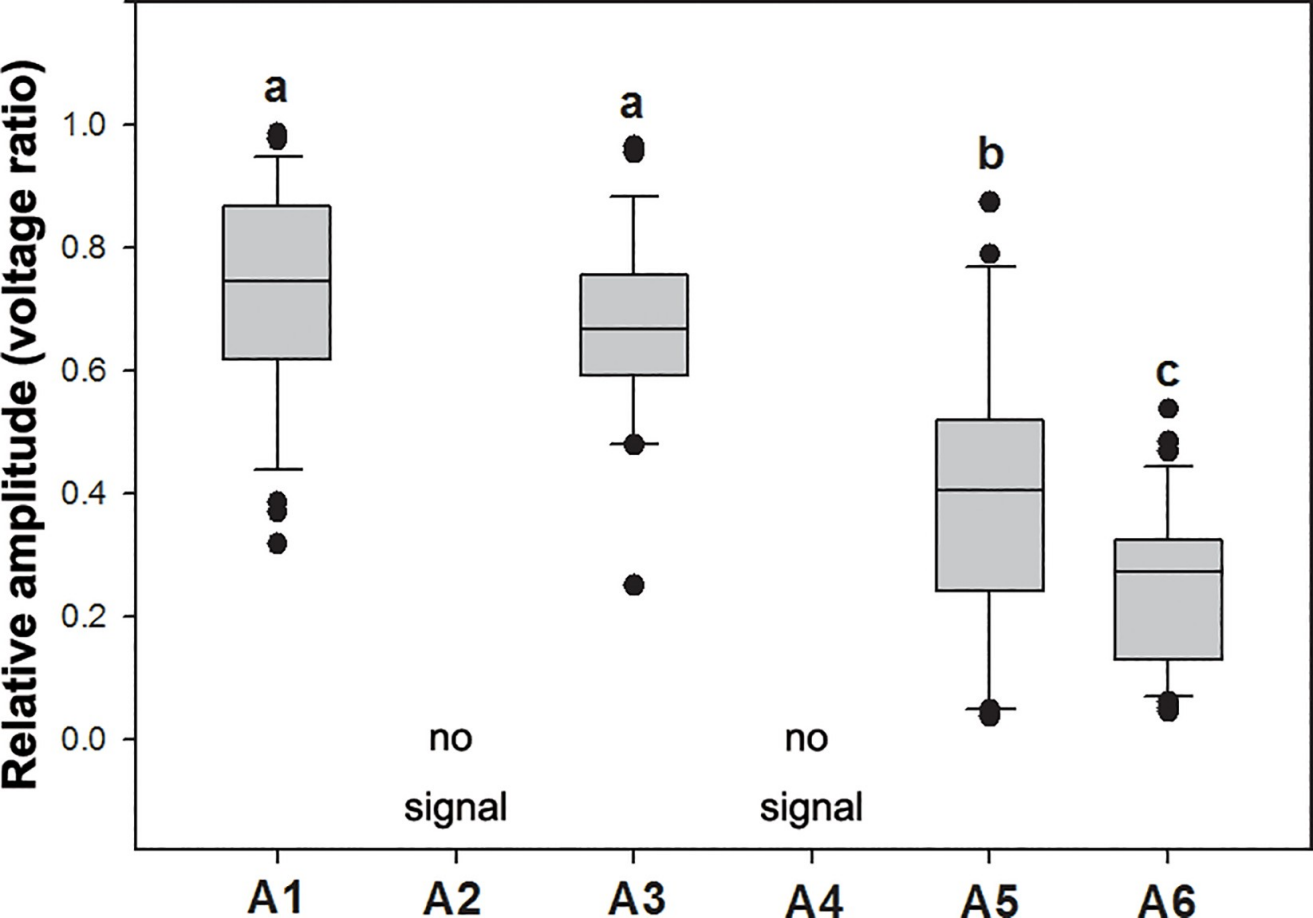
Signal amplitude (voltage ratio) of male calling of *Macrohomotoma gladiata* in each treatment. A1: Control (entire forewing and hindwing, n = 13); A2: Forewing cut (n = 10); A3: Hindwing cut (n = 9); A4: Both wings cut (n = 7); A5: Anal area of forewing cut (n = 7); A6: Forewing cut but axillary sclerite left (n = 11). (Kruskal Wallis test, H = 70.73, *P* < 0.001).

**Fig 4:**
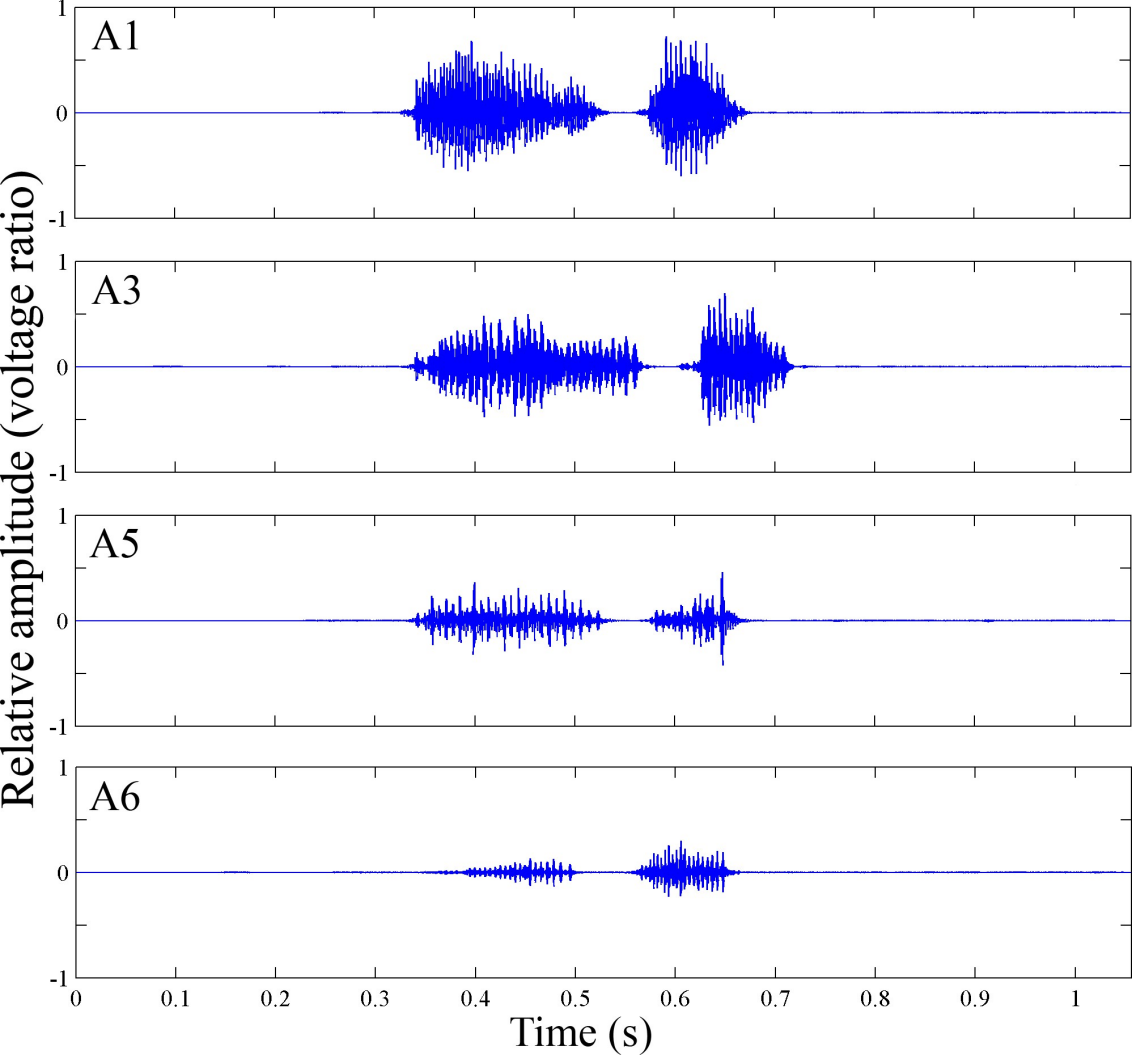
Oscillogram of treated vibrational signals of *Macrohomotoma gladiata* Kuwayama. A1: Control; A3: Hindwing cut; A5: Anal area of forewing cut; A6: Forewing cut but axillary sclerite left.

Among the six treatments, the individuals of A1, A3, A5, and A6 were able to make signals, however, A2 and A4 could not (Fig 3). The signal amplitude of A1 (0.73 ± 0.18) and A3 (0.67 ± 0.15) were significantly higher than that of A5 (0.40 ± 0.23) and A6 (0.25 ± 0.13) (Figs 3 and 4, S3 Table).

The results for the other four species of psyllids, i.e., *Me*. *camphorae*, *T*. *sozanica*, *C*. *oluanpiensis*, and *C*. *tobirae*, were consistent with that of *Ma*. *gladiata*. Individuals of both A1 and A6 could produce signals, however, A2 could not (Table 1, S4 Table). The signal amplitude of member from the A1 treatment was significantly larger than that of those from the A6 treatment (Table 1, S4 Table).

**Table 1:** The effect of forewing cut treatment (A2) and forewing cut but axillary sclerite left treatment (A6) on signal amplitude (voltage ratio) in psyllid species belonging to different families.

Species (Family)	Control treatment (A1)		Forewing cut but axillary sclerite left treatment (A6)		Forewing cut treatment (A2)
	Individual (Calls)	Signal amplitude	Individual (Calls)	Signal amplitude^1^	Signal amplitude
*Mesohomotoma camphorae* (Carsidaridae)	3 (9)	0.08 ± 0.05	3 (9)	0.02 ± 0.10**	no signal
*Trioza sozanica* (Triozidae)	4 (11)	0.56 ± 0.18	4 (12)	0.26 ± 0.16**	no signal
*Cacopsylla oluanpiensis* (Psyllidae)	3 (9)	0.61 ± 0.28	3 (7)	0.21 ± 0.12*	no signal
*Cacopsylla tobirae* (Psyllidae)	4 (10)	0.63 ± 0.21	4 (9)	0.24 ± 0.22**	no signal

^1^The signal amplitude was compared between A1 and A6 on each species using Wilcoxon signed-rank test

* *P* < 0.05

** *P* < 0.01

### The surface of the psyllid signal producing structure

**Fig 5:**
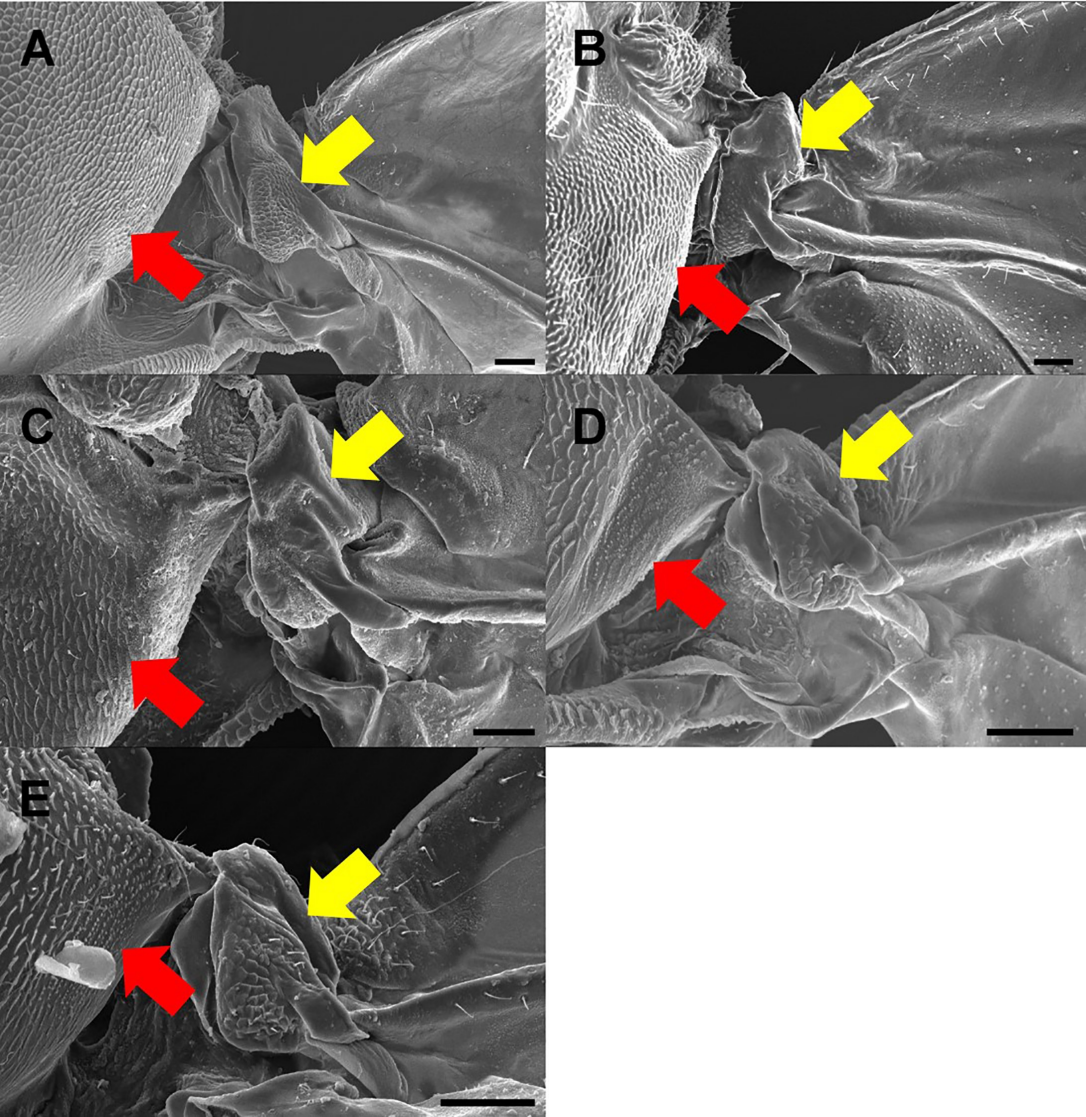
Photographs of scanning electron microscope showing dorsal view of the axillary sclerite in five species of psyllids belonging to different families. A: *Macrohomotoma gladiata* Kuwayama; B: *Trioza sozanica* (Boselli); C: *Mesohomotoma camphorae* Kuwayama; D: *Cacopsylla oluanpiensis* (Yang); E: *Cacopsylla tobirae* (Miyatake). Yellow arrow: Protuberances on the surface of secondary axillary sclerite. Red arrow: scale-like surface of mesothorax scutum.

The previous data indicate that the axillary sclerite is the main component for psyllids to produce signals. SEM images of the dorsal and lateral view of *Ma*. *gladiata*, *Me*. *camphorae*, *T*. *sozanica*, *C*. *oluanpiensis*, and *C*. *tobirae* show that the dorsal surface of secondary axillary sclerite is rough and possesses many protuberances (Fig 5). In addition, the rough surface on the mesothorax appears to form the area of friction with the dorsal surface of the secondary axillary sclerite (Fig 5).

## Discussion

As a result of the wing-cut experiments, we were able to elucidate the crucial relationship between signal production and wings. We ensured that psyllids are not able to emit signals without forewing and it is now clear that the hindwing is certainly not evolved in signal production. The psyllids with only forewings left were able to produce signals, and the signal amplitude of those individuals was not significantly different compared with control treatment (Fig 3; Table 1). According to this evidence, we confirm the importance of the forewing in contributing to the signal producing mechanism of psyllids.

### Hypothesis testing

#### Wingbeat hypothesis

Wingbeats of insects such as mosquitos and bees, can produce rarefaction waves, which are transmitted through the air [36]. Usually the dominant frequency of these signals is consistent with the frequency of the wingbeat [37, 38]. In this study, the dominant frequency of vibrational signals and wingbeat frequency did not match and there is about seven-fold difference. It is also not likely that the dominant frequency we recorded is a seventh harmonic of the fundamental frequency of wingbeat because the amplitude of the signal drops drastically at higher harmonics [39]. Moreover, the major transmitted route for psyllid signals is the substrate, i.e. plant twigs and leaves, not the air. Thus, we confirmed that psyllids could not make signals solely via wingbeats, which means that friction must occur. This hypothesis is rejected.

#### Wing-wing friction hypothesis

Individuals from the A2 treatments were not able to produce signals. This suggests that the hindwing is not involved in signal producing mechanism of psyllids. As well, individuals from the A6 treatments are able to produce signals which also supports rejection of this hypothesis. In addition, through our high-speed video observation (S1 Video and S2 Video), we see that the forewing movement is vertical when psyllids produce signals. Wing-wing friction is likely to be horizontal as seen in signal production of male crickets.

#### Wing-thorax friction hypothesis

This hypothesis suggested that friction between the axillary cord of the thorax and the anal area of the forewing is responsible for signal production. Based on morphological examination, all psyllids have axillary cords on their thorax [21–23]. We have confirmed the importance of the forewing for signal production according to the results from treatments A2, A3, and A4. Further, results of the forewing anal area cut treatment (A5) showed the mean amplitude of signal was half compared with that of the control treatment. This suggests that anal area of forewing plays a considerably important role on signal production. Although this result does not fit the hypothesis completely, it does show the significance of the anal area of the forewing for signal production.

#### Leg-abdomen friction hypothesis

During psyllid calling, we can usually see the upward movement and slight vibration of the abdomen. For example, the abdomen of *T*. *sozanica*, which produces a long call (> 10 seconds), would move upward at the beginning of signal production and slightly vibrate, then move downward to the natural resting position when calling ended. Further, we did not observe obvious rubbing behavior between the leg and abdomen when psyllids produce signals, suggesting that this hypothesis is likely to be invalid.

#### Wing-leg friction hypothesis

Heslop-Harrison [20] thought the wing-leg hypothesis was the minor mechanism of signal producing and that leg-abdomen friction was the major mechanism of signal production in psyllids. However, the leg-abdomen friction and wing-leg hypothesis both were rejected by this study based on the hindwing cut treatment (A3) and forewing cut in combination with the left axillary sclerite treatment (A6), because individuals of these two treatments were able to produce signals.

#### Axillary sclerite-thorax friction hypothesis

This hypothesis involving the axillary sclerite in psyllid signal production was first raised in this study and evolved from the result of the A5 treatment. We originally considered that the anal area of forewing was the major signal producing structure of psyllids but individuals without the anal area of the forewing (A5) were able to make signals. This suggests other structures may involve in signal production. The axillary sclerite of the forewing is a heavily-sclerotized structure that appears to have the potential for producing signals and is the reason for proposing this hypothesis. We noticed that individuals of the forewing cut (A2) and both wings cut treatments (A4) were not able to produce signals; however, individuals with the forewing cut but the axillary sclerite left intact (A6) were able to produce signals suggesting that this theory may have some validity. The amplitude of signals of the forewing cut with axillary sclerite intact (A6) are significantly lower than that of hindwing cut (A3) and control treatments (A1).

The sclerotized structure of the axillary sclerite in psyllids were rough and may be suitable for friction with the thorax to produce signals. Especially the second axillary sclerite that is well-developed structurally and possesses scale-like structure on the surface.

### The signal producing mechanism of psyllids

We conclude that the signal producing mechanism of Psylloidea has two components. One is via the stridulation between anal area of forewing and the axillary cord on mesothorax. The other is via stridulation between the axillary sclerite of forewing and thorax. The finding of the axillary sclerite as a signal producing mechanism is a novel scientific finding. The forewings of psyllids play an important role during signal production but the loss of the hindwing does not affect signal production (Figs 3 and 4). Results showed that the signal amplitude declines if the anal area of forewing was removed or only the axillary sclerite is left intact. These two components may work together in signal production of psyllids. However, the contribution of these two signal producing mechanisms in vibrational behavior of psyllids needs to be examined.

### Signal producing structure and systematics

Taxonomic usefulness of acoustic apparati in insects, is well documented. Villet [40] used several characters of the tymbal organ in his taxonomic revisions, including shape and size of operculum, as well as the meracanthus, and ribs on the tymbal. In Orthoptera, the teeth number on the file of the forewing of crickets are different among species and they serve as diagnostic characters [41]. It is now apparent that the morphology of the signalling apparatus of psyllids may be used in species identification as well. Tishechkin [23] pointed out that the axillary cord is different among psyllids and suggested that axillary cord possesses characteristics distinguishable at the family level. This study has uncovered that the scale-like structure on the axillary sclerite of psyllids can be further measured and compared, and this characteristics appear to be useful taxonomically.

The current accepted phylogeny of Hemiptera suggests that the Sternorrhyncha is an older clade within Hemiptera [42, 43]. Three superfamilies of Sternorrhyncha, Psylloidea, Aphidoidea, and Aleyrodoidea, have various signal producing mechanisms, which may evolved independently as they do not possess a tymbal-like structure for signal production. Members of Auchenorrhyncha and Heteroptera, such as Aphrophoridae, Cicadellidae, Dictyopharidae, Issidae, Alydidae, Cydnidae, Rhopalidae, produce signals using a tymbal [9]. This phenomenon suggests that the tymbal mechanism is a synapomorphic character that evolved after Sternorrhyncha branched off. The signal producing mechanism of Psylloidea confirmed by this study may be an apomorphy for this group. This finding provides evidence of the potential monophyly of Psylloidea. Also, we found that axillary sclerites were different in scale pattern among species under SEM and this structure may have the potential to be taxonomically useful at the species level. Quantitative analysis for characteristics of the axillary sclerite could be further conducted for potential use in delineating the higher classification of Psylloidea.

## Supporting information

S1 Fig Illustration of wing veins of *Macrohomotoma gladiata* Kuwayama. (JPG)

S1 Table Collecting information of psyllid specimens in this study. (DOCX)

S2 Table The wingbeat frequency of calling behavior of male *Macrohomotoma gladiata* Kuwayama. (DOCX)

S3 Table The relative amplitude (voltage ratio) of signal of *Macrohomotoma gladiata* Kuwayama in different wing-cut treatments. (DOCX)

S4 Table The relative amplitude (voltage ratio) of signal of four psyllid species in A1 and A6 treatments. (DOCX)

S1 Audio Vibrational duets of male and female of *Macrohomotoma gladiata* Kuwayama. (MP3)

S2 Audio Vibrational signal of hind wing cut A3 treatment. (MP3)

S3 Audio Vibrational signal of anal area cut A5 treatment. (MP3)

S4 Audio Vibrational signal of axillary sclerite left A6 treatment. (MP3)

S1 Video Vibrational behavior of *Macrohomotoma gladiata* Kuwayama 80x slow motion. (MP4)

S2 Video Vibrational behavior of *Macrohomotoma gladiata* Kuwayama 10x slow motion. (MP4)
