# Supplementary figures and images for: Vibrational behavior of psyllids (Hemiptera: Psylloidea): Functional morphology and mechanisms
Source: PLoS One. 2019 Sep 11;14(9):e0215196. doi: 10.1371/journal.pone.0215196 (PMC6738581; doi:10.1371/journal.pone.0215196)

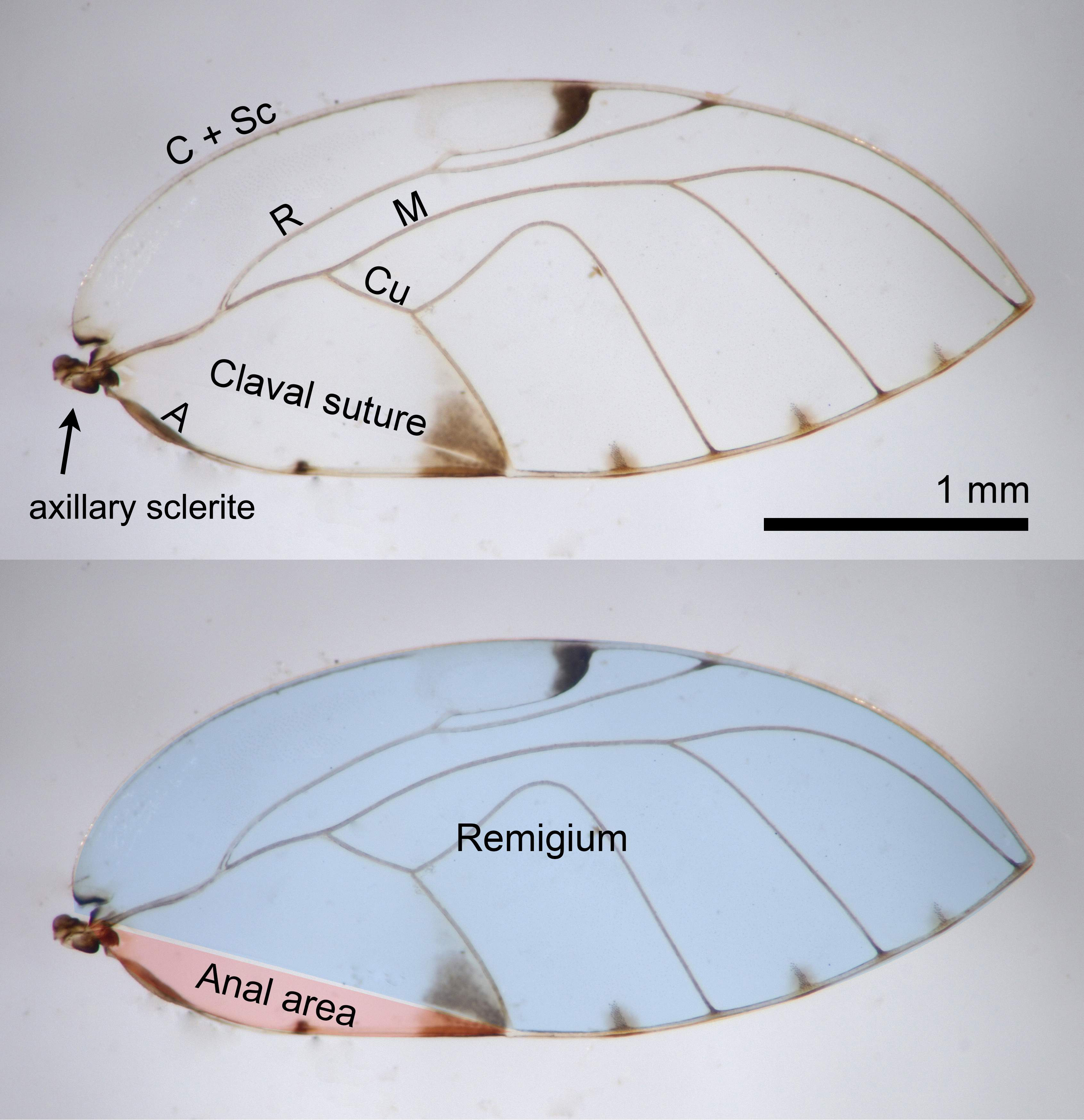

Supplement: S1 Fig — (JPG) [file pone.0215196.s001.jpg]
