# Supplementary material for: Vibrational behavior of psyllids (Hemiptera: Psylloidea): Functional morphology and mechanisms
Source: PLoS One. 2019 Sep 11;14(9):e0215196. doi: 10.1371/journal.pone.0215196 (PMC6738581; doi:10.1371/journal.pone.0215196)
Supplement: S1 Table — (DOCX) [file pone.0215196.s002.docx]

**S1 Table. Collecting information of psyllid specimens in this study**

| **Psyllid species** | **Family** | **Location** | **Coordinate** | **Date** |
| --- | --- | --- | --- | --- |
| ***Macrohomotoma gladiata* Kuwayama** | Homotomidae | Taichung City, Dali Dist. | 24° 05' 17" N 120° 39' 45" E | 2013. I 2014.VI |
| ***Trioza sozanica* (Boselli)** | Triozidae | Nantou County, Ren’ai Township | 24° 05' 13" N 121° 01' 35" E | 2013. X |
|  |  | Taichung City, Taiping Dist. | 24° 10' 46" N 120° 56' 10" E | 2014. II |
| ***Mesohomotoma camphorae* Kuwayama** | Carsidaridae | Taitung County, Taitung City | 22° 42' 10" N 121° 04' 52" E | 2014. I |
|  |  | Taichung City, South Dist. | 24°07'14" N 120°40'27" E | 2014. III |
| ***Cacopsylla oluanpiensis* (Yang)** | Psyllidae | Taichung City, Houli Dist. | 24° 19' 41" N 120° 42' 56" E | 2013. XI 2013. XII |
| ***Cacopsylla tobirae* (Miyatake)** | Psyllidae | Hsinchu County, Baoshan Township | 24° 44' 39" N 121° 02' 17" E | 2013. XI |
