# Supplementary material for: Vibrational behavior of psyllids (Hemiptera: Psylloidea): Functional morphology and mechanisms
Source: PLoS One. 2019 Sep 11;14(9):e0215196. doi: 10.1371/journal.pone.0215196 (PMC6738581; doi:10.1371/journal.pone.0215196)
Supplement: S2 Table — (DOCX) [file pone.0215196.s003.docx]

**S2 Table. The wingbeat frequency of calling behavior of male *Macrohomotoma gladiata* Kuwayama**

| Calls | Duration of first chirp | Number of wingbeat in 10x slow motion video | The wingbeat frequency of first chirp | Duration of second chirp | Number of wingbeat in 10 x slow motion video | The wingbeat frequency of second chirp |
| --- | --- | --- | --- | --- | --- | --- |
| 1 | 1.98 | 24 | 121.21 | 1.03 | 15 | 145.63 |
| 2 | 1.93 | 24 | 124.35 | 1.11 | 16 | 144.14 |
| 3 | 2.04 | 25 | 122.55 | 1.05 | 15 | 142.86 |
| 4 | 2.12 | 24 | 113.21 | 1.04 | 15 | 144.23 |
| 5 | 2.22 | 26 | 117.12 | 1.12 | 15 | 133.93 |
| 6 | 2.49 | 33 | 132.53 | 1.14 | 16 | 140.35 |
| 7 | 2.45 | 28 | 114.29 | 1.16 | 16 | 137.93 |
| 8 | 2.32 | 27 | 116.38 | 1.19 | 16 | 134.45 |
| 9 | 2.17 | 27 | 124.42 | 1.08 | 15 | 138.89 |
| 10 | 2.37 | 26 | 109.70 | 1.11 | 16 | 144.14 |
| 11 | 2.32 | 27 | 116.38 | 1.15 | 16 | 139.13 |
| 12 | 2.28 | 27 | 118.42 | 1.27 | 16 | 125.98 |
| 13 | 2.43 | 28 | 115.23 | 1.19 | 16 | 134.45 |
| 14 | 2.31 | 28 | 121.21 | 1.07 | 15 | 140.19 |
| 15 | 2.48 | 31 | 125.00 | 1.08 | 15 | 138.89 |
| 16 | 2.27 | 28 | 123.35 | 1.09 | 16 | 146.79 |
| 17 | 2.57 | 29 | 112.84 | 1.08 | 15 | 138.89 |
| 18 | 2.59 | 28 | 108.11 | 1.19 | 17 | 142.86 |
| 19 | 2.38 | 28 | 117.65 | 1.13 | 16 | 141.59 |
| 20 | 2.36 | 29 | 122.88 | 1.13 | 16 | 141.59 |
| 21 | 2.53 | 29 | 114.62 | 1.16 | 16 | 137.93 |
| 22 | 2.34 | 29 | 123.93 | 1.20 | 16 | 133.33 |
| 23 | 2.39 | 29 | 121.34 | 1.13 | 16 | 141.59 |
| 24 | 2.65 | 31 | 116.98 | 1.08 | 15 | 138.89 |
| 25 | 2.55 | 30 | 117.65 | 1.11 | 16 | 144.14 |
| 26 | 2.48 | 30 | 120.97 | 1.12 | 16 | 142.86 |
| 27 | 2.64 | 30 | 113.64 | 1.11 | 16 | 144.14 |
| 28 | 2.58 | 29 | 112.40 | 1.14 | 16 | 140.35 |
| 29 | 2.41 | 28 | 116.18 | 1.13 | 16 | 141.59 |
| 30 | 1.97 | 26 | 131.98 | 1.17 | 16 | 136.75 |
| 31 | 2.70 | 30 | 111.11 | 1.21 | 17 | 140.50 |
| 32 | 2.65 | 30 | 113.21 | 1.13 | 16 | 141.59 |
| 33 | 2.51 | 30 | 119.52 | 1.19 | 17 | 142.86 |
| 34 | 2.49 | 28 | 112.45 | 1.18 | 16 | 135.59 |
