# Supplementary material for: Vibrational behavior of psyllids (Hemiptera: Psylloidea): Functional morphology and mechanisms
Source: PLoS One. 2019 Sep 11;14(9):e0215196. doi: 10.1371/journal.pone.0215196 (PMC6738581; doi:10.1371/journal.pone.0215196)
Supplement: S3 Table — (DOCX) [file pone.0215196.s004.docx]

**S3 Table. The relative amplitude (voltage ratio) of signal of *Macrohomotoma gladiata* Kuwayama in different wing-cut treatments**

| Treatments | A1 | A3 | A5 | A6 |
| --- | --- | --- | --- | --- |
|  | 0.32 | 0.05 | 0.54 | 0.85 |
|  | 0.46 | 0.05 | 0.48 | 0.74 |
|  | 0.39 | 0.04 | 0.47 | 0.59 |
|  | 0.74 | 0.40 | 0.33 | 0.73 |
|  | 0.72 | 0.20 | 0.25 | 0.67 |
|  | 0.78 | 0.60 | 0.36 | 0.80 |
|  | 0.98 | 0.87 | 0.18 | 0.64 |
|  | 0.71 | 0.39 | 0.30 | 0.71 |
|  | 0.37 | 0.79 | 0.11 | 0.70 |
|  | 0.82 | 0.46 | 0.28 | 0.68 |
|  | 0.95 | 0.36 | 0.27 | 0.60 |
|  | 0.98 | 0.55 | 0.31 | 0.59 |
|  | 0.93 | 0.28 | 0.17 | 0.74 |
|  | 0.84 | 0.29 | 0.13 | 0.59 |
|  | 0.89 | 0.35 | 0.16 | 0.64 |
|  | 0.84 | 0.43 | 0.28 | 0.49 |
|  | 0.88 | 0.48 | 0.33 | 0.25 |
|  | 0.95 | 0.69 | 0.32 | 0.64 |
|  | 0.83 | 0.42 | 0.18 | 0.64 |
|  | 0.98 | 0.19 | 0.32 | 0.62 |
|  | 0.85 | 0.40 | 0.32 | 0.48 |
|  | 0.49 |  | 0.13 | 0.96 |
|  | 0.47 |  | 0.08 | 0.76 |
|  | 0.66 |  | 0.13 | 0.86 |
|  | 0.60 |  | 0.28 | 0.95 |
|  | 0.75 |  | 0.40 | 0.75 |
|  | 0.70 |  | 0.41 | 0.48 |
|  | 0.65 |  | 0.19 |  |
|  | 0.59 |  | 0.06 |  |
|  | 0.71 |  | 0.05 |  |
|  | 0.87 |  | 0.10 |  |
|  | 0.74 |  | 0.10 |  |
|  | 0.67 |  | 0.05 |  |
|  | 0.44 |  |  |  |
|  | 0.84 |  |  |  |
|  | 0.74 |  |  |  |
|  | 0.85 |  |  |  |
|  | 0.88 |  |  |  |
|  | 0.62 |  |  |  |
