# Supplementary material for: Vibrational behavior of psyllids (Hemiptera: Psylloidea): Functional morphology and mechanisms
Source: PLoS One. 2019 Sep 11;14(9):e0215196. doi: 10.1371/journal.pone.0215196 (PMC6738581; doi:10.1371/journal.pone.0215196)
Supplement: S4 Table — (DOCX) [file pone.0215196.s005.docx]

**S4 Table. The relative amplitude (voltage ratio) of signal of four psyllid species in A1 and A6 treatments**

| Species | Treatments |  |  |  |  |  |  |  |  |  |  |  |
| --- | --- | --- | --- | --- | --- | --- | --- | --- | --- | --- | --- | --- |
| *Mesohomotoma camphorae* | A1 | 0.06 | 0.07 | 0.06 | 0.02 | 0.04 | 0.02 | 0.18 | 0.13 | 0.12 |  |  |
|  | A6 | 0.03 | 0.01 | 0.03 | 0.01 | 0.01 | 0.01 | 0.03 | 0.03 | 0.03 |  |  |
| *Trioza sozanica* | A1 | 0.84 | 0.87 | 0.68 | 0.45 | 0.53 | 0.63 | 0.36 | 0.49 | 0.41 | 0.58 |  |
|  | A6 | 0.05 | 0.07 | 0.10 | 0.29 | 0.45 | 0.35 | 0.47 | 0.41 | 0.41 | 0.11 | 0.21 |
| *Cacopsylla oluanpiensis* | A1 | 0.83 | 0.86 | 0.94 | 0.32 | 0.39 | 0.47 | 0.79 | 0.73 | 0.16 |  |  |
|  | A6 | 0.36 | 0.40 | 0.16 | 0.13 | 0.22 | 0.13 | 0.09 |  |  |  |  |
| *Cacopsylla tobirae* | A1 | 0.37 | 0.65 | 0.49 | 0.39 | 0.84 | 0.69 | 0.78 | 0.91 | 0.39 | 0.84 |  |
|  | A6 | 0.04 | 0.05 | 0.03 | 0.53 | 0.55 | 0.32 | 0.06 | 0.46 | 0.13 |  |  |
